# A cross sectional study exploring the relationship of self-reported physical activity with function, kinesiophobia, self-efficacy and quality of life in an Asian population seeking care for knee osteoarthritis

**DOI:** 10.1186/s12891-024-07181-y

**Published:** 2024-01-18

**Authors:** Anthony J. Goff, Lester E. Jones, Chien Joo Lim, Bryan Yijia Tan

**Affiliations:** 1 Singapore Institute of Technology, Health and Social Sciences, 10 Dover Drive, Singapore, 138683 Singapore; 2 Judith Lumley Centre, La Trobe University, Plenty Rd & Kingsbury Dr, Bundoora, Vic 3086 Australia; 3 Orthopaedic Surgery, Woodlands Health, Yishun Community Hospital, 2 Yishun Central 2 Tower E, Singapore, 768024 Singapore

**Keywords:** Physical activity, Knee, Osteoarthritis, Self-efficacy, Kinesiophobia, Quality of life, Exercise

## Abstract

**Background:**

Physical activity is a guideline-recommended first-line intervention for people with knee osteoarthritis. Physical activity levels, and its potential correlates, is underexplored in Asian populations with knee osteoarthritis.

**Methods:**

Participants enrolled in a longitudinal study in Singapore self-reported physical activity (UCLA activity score), function (Knee Osteoarthritis Outcome Score [KOOS-12]), kinesiophobia (Brief fear of movement [BFOM]), self-efficacy (ASES-8), and quality of life (EQ-5D-5 L). One-Way ANOVA was used to test the difference in outcomes between UCLA categories, while ordinal logistic regression was used to identify the associated factors to physical activity level.

**Results:**

Seventy-three percent of all enrolled participants (*n* = 311/425) reported either inactivity or low physical activity (median 4, IQR 3–5). Significant, weak, positive correlations were observed be-tween UCLA activity score and either KOOS-12 (Spearman’s rho: 0.1961; *p* < 0.001), ASES-8 (0.1983; *p* = 0.004), or EQ-5D-5 L (0.2078; *p* < 0.001). A significant, weak, negative correlation was observed between physical activity and BFOM (-0.2183; *p* < 0.001). Significant differences in function between groups (moderate vs. inactive or low physical activity) were not clinically important. Participants with obesity, from the eldest age category (i.e. ≥75), or who identified as Malay or female, were less physically active than those with a healthy BMI, below the age of 54, or who identified as Chinese or male, respectively.

**Conclusion:**

Healthcare professionals in Asia should be aware of the large proportion of people with knee osteoarthritis who are either inactive or have low physical activity levels. Screening for, and offering interventions to promote, physical activity and its correlates should be prioritised.

**Supplementary Information:**

The online version contains supplementary material available at 10.1186/s12891-024-07181-y.

## Background

Knee osteoarthritis is a leading cause of disability worldwide [1], with symptomatic knee osteoarthritis affecting up to one in ten people over the age of 50 in Asia [2]. Personal and societal burdens related to knee osteoarthritis are predicted to rise significantly in the next decade, in part due to ageing populations, but also due to increased levels of obesity and physical inactivity/sedentary lifestyles [3, 4].

Physical inactivity for adults and older adults is defined as a failure to complete 150 min of moderate to vigorous physical activity across five or more days [5]. Physical inactivity is a modifiable risk factor for the development [6, 7] and progression [8, 9] of knee osteoarthritis. Conversely, physical activity is effective at reducing pain, and increasing function, performance and health related quality of life for people with knee osteoarthritis [10, 11]. Consequently, all major clinical practice guidelines for people with knee osteoarthritis advocate physical activity as first-line care alongside education, exercise therapy and, when appropriate, weight management [12–15]. Despite this, a recent systematic review estimated that just 13% of people with knee osteoarthritis meet current international physical activity guidelines [16]. However, it is worth noting that this review was heavily influenced by research in Western countries, with just 2 of the 27 included studies being performed in Asia [17, 18]. Further, both of these studies were performed in small, female only populations in Japan, representing just 55 participants out of the 3266 included. As such, further research is required to ascertain whether these estimates of physical activity/inactivity are representative of Asian populations with knee osteoarthritis outside of Japan.

Previous research has identified a number of factors that influence engagement with physical activity for people with knee osteoarthritis, namely increased age, non-white ethnicity, increased osteoarthritis symptoms, and female gender [19]. However, these findings are also informed primarily from trials performed in Western populations with just 2 of the 29 trials being performed in Asia (Japan) [20, 21]. Other factors such as kinesiophobia [22] and self-efficacy [23] may also contribute towards lower physical activity levels of people with knee osteoarthritis and are again underexplored in Asian populations. Research is therefore urgently needed to better understand physical activity and its potential correlates for Asian populations with knee osteoarthritis. Identification of this will help to inform targeted interventions to increase physical activity for people with knee osteoarthritis and facilitate subsequent improvements in patient outcomes, health and quality of life.

This study aims to identify the self-reported physical activity levels of a multi-cultural Asian group of people seeking care for knee osteoarthritis. We also aim to explore the relationship of self-reported physical activity with self-reported function, kinesiophobia, self-efficacy and quality of life.

## Methods

### Study design

This study analyzed baseline data collected as part of a pre-registered (clinicaltrials.gov, NCT04942236 first registered 28/06/2021) multi-center, prospective cohort study for people with knee osteoarthritis in Singapore [24]. This study is reported following the STROBE guidelines [25].

### Ethical approval

Ethical approval was provided by the National Healthcare Group Domain Specific Review Board in Singapore (NHG DSRB; Reference number: WHC/2020-00076).

### Participants and recruitment

Participants were recruited to the main multi-center, prospective cohort study between July 2020 and January 2022 when they presented for treatment at either the orthopedic or physiotherapy clinics at hospitals within the National Healthcare Group of Singapore (Tan Tock Seng Hospital and Khoo Teck Puat Hospital). Participants were eligible for the study if they met the NICE clinical diagnostic criteria for knee osteoarthritis [13] (i.e., they were i.  are aged 45 or over, ii. have activity-related joint pain and, iii. have either no morning joint-related stiffness or morning stiffness that lasts no longer than 30 minutes) and they were independent community mobilisers (with or without walking aids). Participants were excluded if they had an alternative diagnosis for their knee symptoms (e.g., referred pain from hip/spine), had secondary arthritis (e.g., inflammatory), were unable to comply with the study protocol (e.g., significant cognitive impairment) or had severe medical comorbidities impairing activities of daily living (e.g., COPD on long-term oxygen therapy, cardiac failure with significantly impaired effort tolerance, stroke with significant residual functional weakness). Those who had received a previous knee arthroplasty, were wheelchair bound or who were pregnant, were also excluded from participating in the study. Potentially eligible participants were initially identified by pre-screening the relevant clinic appointment lists and/or accessing patients’ e-medical records. In an attempt to reduce persuasion from the participants primary care giver at the appointment, eligible participants were then approached by a study coordinator during the clinic visit to share and explain details about the study. Written consent was obtained from interested participants prior to enrolment.

Upon enrollment, participants provided demographic details and completed a number of self-reported outcome measures using either a hard copy form during the clinic visit itself, or via a self-administered online form (FormSG), which the participant completed at their own convenience. Demographic details included age, gender, ethnicity, Body Mass Index (BMI), employment status, education level and whether they presented with unilateral or bilateral knee symptoms.

### Outcomes

This study used baseline scores for the Knee Osteoarthritis Outcome Score (KOOS-12) [26], UCLA activity scale (1-10 version) [27], Arthritis Self-Efficacy Scale (ASES-8) [28], Brief Fear of Movement (BFOM) questionnaire [29] and the EQ-5D-5L [30]. These outcomes were selected primarily due to their frequent use in research for people with knee osteoarthritis [29, 31–36], but also due to the availability of valid and reliable English and Chinese versions of the outcomes [37–41]. The default language for outcomes was English, however, we used the Chinese versions when necessary (i.e. the participant did not speak English).  The KOOS-12 is a 12-item, 4-domain assessment tool measuring participants’ perception of their knee function. Domains include pain, function and daily living, and quality of life, and are measured using 5-point Likert scales from 0 to 4, with 4 questions per domain [26]. Questions in each domain are used to calculate summative scores, with higher scores indicating more optimal outcomes. The UCLA activity score is a scale that assesses the self-reported physical activity level of a participant based upon 10 descriptive activity levels [27]. Higher scores indicate increased levels of physical activity. The ASES-8 assesses participant’s confidence in performing certain daily tasks [28]. Summative scores indicate the level of self-efficacy the participant has in managing their arthritis, with higher scores indicating higher levels of self-efficacy. The BFOM questionnaire assesses the fear of movement that the participant experiences [29]. The questionnaire consists of six questions with a 4-point Likert scale ranging from 1 (strongly disagree) to 4 (strongly agree). A summative score of the six questions is computed, with higher score indicating greater fear of movement. The EQ-5D-5L questionnaire is commonly used to assess quality of life and consisting of 5 domains (mobility, self-care, usual activities, pain/discomfort, and anxiety/depression) [30]. Each dimension contains 5 levels, from no problem to extreme problems. Relevant permission was granted for the use of the EQ-5D-5L and we used generated utility values for the outcome based on the Singapore value set provided by previous research [42].

### Sample size

Sample size was calculated using STATA version 14.0 (STATACorp. 2015. Stata Statistical Software: Released 14. College Station, TX: StataCorp LP). As a weak correlation has been observed between the UCLA activity score and the KOOS-12 [43], sample size was estimated based on a correlation coefficient of 0.15 to produce a sample size that would be powered enough to detect even weak correlations between UCLA and KOOS-12, ASES-8, BFOM and EQ-5D-5L [44]. We therefore required data for a minimum of 347 participants considering 0.05 type I error and 80% power of study.

### Data analysis and representation

At the time of data analysis, all recruited participants to the main longitudinal cohort study were included in data analysis, and complete case analysis was used as the missing data percentage was low and ignorable (<2.8%). Participant demographic and outcome measure responses were de-identified, given a unique identifier number and stored on a secure web-based application widely used for clinical data management in research (Research Electronic Data Capture [REDCAP]). Data were cleaned and analyzed by one member of the research team (LCJ), using SPSS version 26.0. The distribution of continuous variables were checked using skewness, kurtosis and histogram, and data were deemed to be normally distributed with skewness and kurtosis of between -2 and 2, as well as an approximately bell shaped histogram.

Descriptive statistics were used to summarize the participant demographics and raw UCLA activity scores (presented as median, 25^th^ percentile; 75^th^ percentile due to the ordinal nature of the outcome) and for determining correlations with KOOS-12, ASES-8, BFOM and EQ-5D-5L using Spearman’s correlation tests. In alignment with previous research for people with knee osteoarthritis [45], UCLA activity scores were also categorized to one of the following five domains; inactive (1-2), low physical activity level (3-4), moderate physical activity level (5-6), high physical activity level (7-8) and, very high activity level (9-10). The proportion (%) of people categorized to each level of physical activity was calculated, plus the difference in KOOS-12, ASES-8, BFOM and EQ-5D-5L between patients with different physical levels were tested using One Way ANOVA. Post-hoc pairwise comparison with Bonferroni or Dunnet C correction following the significance of One Way ANOVA was used to determine where the significant difference truly came from. Statistical significance was denoted as *p*< 0.05 throughout. Mean difference (MD) between different physical activity levels was compared against previously published Minimum Clinically Important Differences (MCID) for the KOOS-12 (MCID = 11.1) [46].

Ordinal logistic regression analyses were performed to identify factors associated with UCLA activity score. In line with previous research [47, 48], we used BMI categories specific to Asian populations for regression analyses (Healthy <23kg/m [2], Overweight 23-27.4kg/m^2^, Obesity ≥27.5 kg/m^2^) [49]. Univariable analysis was used to determine significant factors associated with physical activity. Variables with a *p* value less than 0.200 in the univariable analysis [50] were included into the multivariable model using stepwise variable selection process with ‘xi’ command from STATA package to expand the categorical variables into indicator variables. Multicollinearity of the final model was also tested. Finally, cross tabulation analyses were performed (either Fisher extract or Pearson’s Chi Squared test) to check for the association between categorical variables. The assumptions for various tests were checked before proceeding to the respective analysis to ensure they were fulfilled, and statistical significance was denoted as *p*< 0.05 throughout.

## Results

Four hundred and twenty-five participants were enrolled in our study. Availability of data for each outcome, plus participant demographics are presented in Table 1. Mean age of participants was 63.59 (standard deviation [SD] 7.97) and BMI was 26.77 kg/m^2^ (SD 5.11). Median UCLA activity score was 4 (inter quartile range [IQR] 3-5). The number and proportion of participants for each category of the 1-10 UCLA activity score are presented in Table 2. See Additional file 1 (Table S1) for more details.

**Table 1:** Participant demographics

Outcome	*n*= (%) in relation to all participants
Category	*n*= (%) for each category of outcome
**All**	**425 (100.0)**
**Age in years**	**425 (100.0)**
-≤ 54	57 (13.4)
-55-64	185 (43.5)
-65-74	142 (33.4)
-≥75	41 (9.7)
**Gender**	**424 (99.8)**
-Female	290 (68.4)
-Male	134 (31.6)
**BMI**	**413 (97.2)**
-Healthy - <23 kg/m^2^	85 (20.0)
-Overweight - 23-27.4 kg/m^2^	191 (44.9)
-Obesity - ≥ 27.5 kg/m^2^	137 (32.2)
**Ethnicity**	**425 (100.0)**
-Chinese	333 (78.35)
-Indian	43 (10.12)
-Malay	42 (9.88)
-Others	7 (1.65)
**Employment status**	**419 (98.6)**
-Employed	236 (56.3)
-Unemployed	19 (4.5)
-Homemaker	48 (11.5)
-Retired	116 (27.7)
**Education level**	**425 (100)**
-Informal	16 (3.8)
-Primary	64 (15.1)
-Secondary	214 (50.4)
-Diploma	64 (15.1)
-University	51 (12.0)
-Others	16 (3.8)
**Presence of symptomatic osteoarthritis in:**	**424 (99.8)**
-Left knee	89 (21.0)
-Right knee	154 (36.3)
-Both knees	181 (42.6)

*BMI *Body Mass Index

**Table 2:**
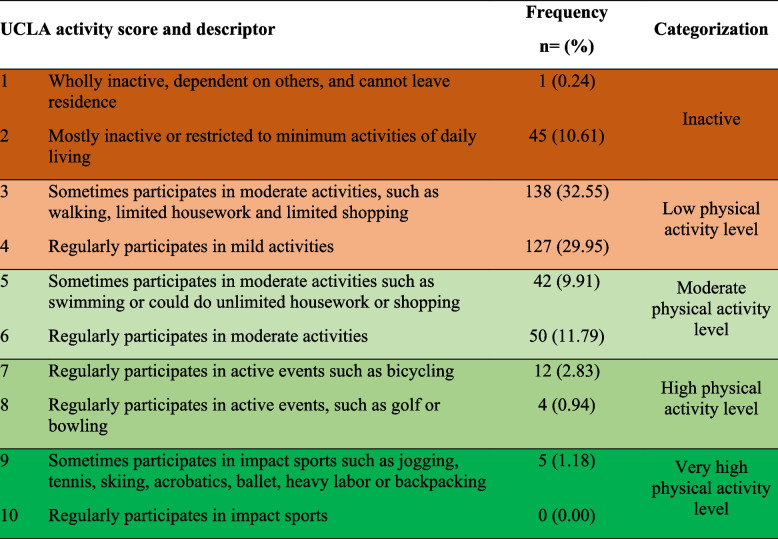
Number and proportion(%) of participants in each category of the 1-10 UCLA activity score

### Correlates of raw (0-10) UCLA activity score to self-reported function, kinesiophobia, self-efficacy and quality of life

**Fig. 1:**
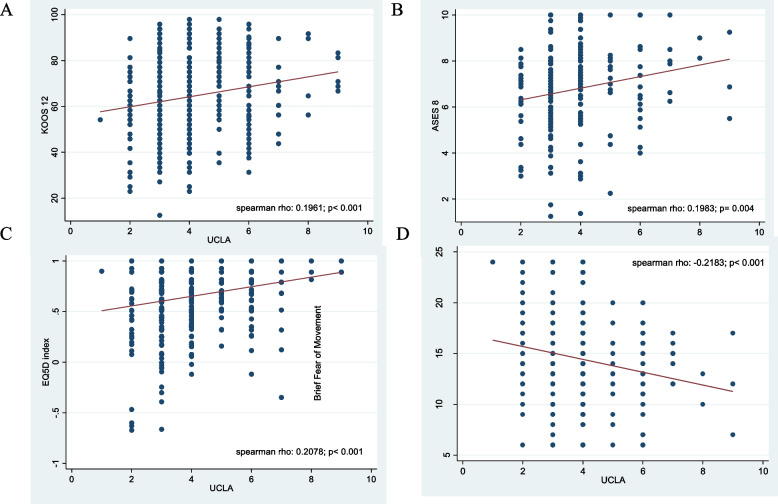
Correlation between linear UCLA Activity Score with KOOS-12 (**A**), ASES-8 (**B**), EQ-5D-5L (**C**) and BFOM (**D**). KOOS-12 = Knee Osteoarthritis Outcome score, ASES-8 = Arthritis Self-efficacy Scale, EQ-5D-5L = measure of quality of life, UCLA = UCLA activity score

Significant, weak, positive correlations were observed between UCLA activity score and KOOS-12 (Spearman’s rho: 0.1961; *p*< 0.001), ASES-8 (0.1983; *p*= 0.004) and EQ-5D-5L (0.2078; *p*< 0.001). A significant, weak, negative correlation was observed between UCLA and the BFOM (-0.2183; *p*< 0.001). Refer to Fig. 1 (A-D) for more details.

### Categorization of UCLA activity score

**Fig. 2:**
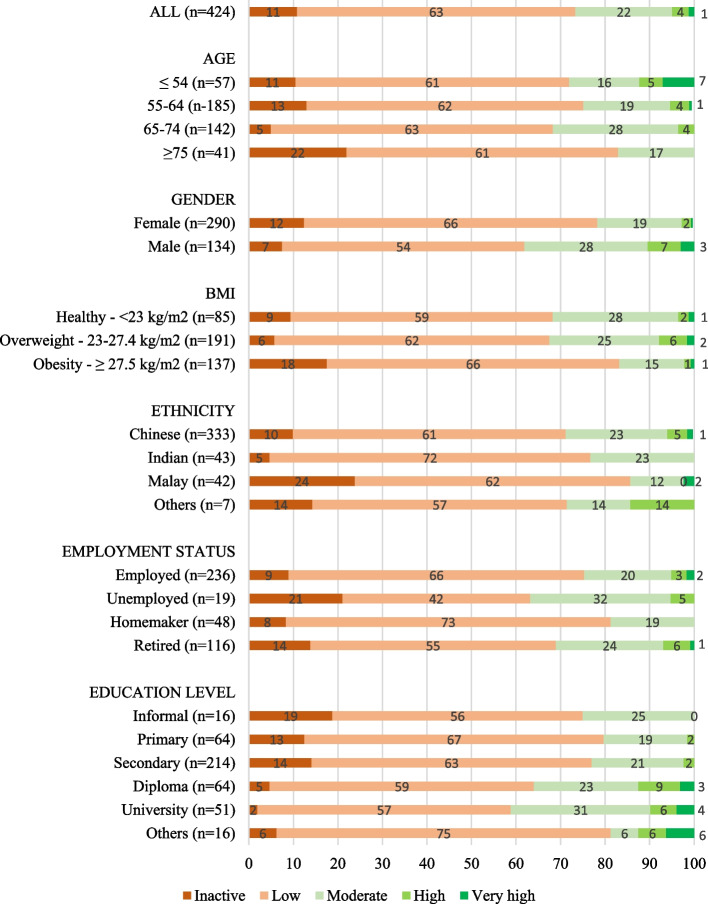
Proportion (%) of people with knee osteoarthritis being categorized as inactive or having low, moderate, high or very high physical activity levels using the UCLA activity scor BMI = Body Mass Index, OA = Osteoarthritis, Inactive = 1-2 on UCLA activity score, Low = 3-4 on UCLA activity score, Moderate = 5-6 on UCLA activity score, High = 7-8 on UCLA activity score, Very high = 9-10 on UCLA activity scoree

The proportion of people with knee osteoarthritis being classified as inactive or having low, moderate, high or very high physical activity levels are presented in Fig. 2. Most participants self-reported low physical activity levels (*n*=265/425, 62.35%).

### Correlates of categorized UCLA activity score to self-reported function, kinesiophobia, self-efficacy and quality of life

Comparisons of mean and standard deviation scores between differing UCLA activity score categorizations are presented in Table 3. Significant differences were observed between physical activity level and KOOS-12, EQ-5D-5L and BFOM (*p*< 0.05). Post-hoc pairwise comparison with Bonferroni correction was performed for KOOS-12 and for BFOM. For KOOS-12, participants with moderate physical activity level had significantly higher, but clinically unimportant differences (i.e., MD = <11.1 [46]), in KOOS-12 than those who are either inactive (mean difference [MD], 95% CI: 10.19, 2.27 to 18.16) or had low physical activity level (MD, 95% CI; 6.22, 0.87 to 11.56). For BFOM, participants with moderate physical activity level had significantly lower BFOM scores compared to inactive participants (MD, 95% CI: -3.18, -5.79 to -0.57). Post-hoc pairwise comparison with Dunnett C was performed for EQ-5D-5L as the homogeneity of variance was not met. For EQ-5D-5L, participants with moderate physical activity levels had significantly higher EQ-5D-5L compared to those who were either inactive (MD, 95% CI: 0.24, 0.05 to 0.44), or had low physical activity levels (MD, 95% CI: 0.10, 0.01 to 0.19). Participants with very high physical activity level had a significantly higher EQ-5D-5L compared to those who were inactive (MD, 95% CI: 0.42, 0.22 to 0.62), or who had low (MD, 95% CI: 0.27, 0.17 to 0.38) or moderate physical activity levels (MD, 95% CI: 0.17, 0.05 to 0.29). See Additional file 1 (Tables S2, S3, S4) for more details.

**Table 3:** Mean and standard deviation of self-reported outcomes physical activity categorization

UCLA activity categorization^a^						
Outcome	Inactive	Low	Moderate	High	Very High	*P* value
KOOS-12	58.92 ± 15.45	62.89 ± 15.63	69.11 ± 16.22	67.32 ± 14.59	74.17 ± 7.60	0.001
ASES-8	6.22 ± 1.68	6.72 ± 1.77	7.08 ± 1.96	8.05 ± 1.21	7.21 ± 1.90	0.103
EQ-5D-5L	0.49 ± 0.43	0.64 ± 0.30	0.74 ± 0.25	0.68 ± 0.37	0.91 ± 0.05	<0.001
BFOM	16.39 ± 4.36	14.62 ± 4.10	13.21 ± 3.94	13.78 ± 2.22	12.00 ± 5.00	0.010

*KOOS *Knee Osteoarthritis Outcome Score, *ASES *Arthritis self-efficacy scale, *BFOM *Brief fear of movement

^a^Inactive = 1-2 on UCLA activity score, Low = 3-4 on UCLA activity score, Moderate = 5-6 on UCLA activity score, High = 7-8 on UCLA activity score, Very high = 9-10 on UCLA activity score.

### Regression analyses and cross tabulation

Ordinal logistic regression analyses of physical activity are presented in Table 4. In summary, participants with obesity, from the eldest age category (i.e. ≥75), or who identified as Malay or female, were less physically active compared to those who had a healthy BMI (adj OR, 95% CI: 0.47, 0.30 to 0.74), were below the age of 54 (adj OR, 95% CI: 0.29, 0.14 to 0.60), or who identified as Chinese (adj OR, 95% CI: 0.44, 0.22 to 0.88) or male (adj OR, 95% CI: 0.46, 0.29 to 0.73), respectively. Additionally, those with high KOOS12 score (adj OR, 95% CI: 1.98, 1.26 to 3.13), or had a diploma (adj OR, 95% CI: 1.85, 1.05 to 3.27) or university level education (adj OR, 95% CI: 2.26, 1.23 to 4.15) had higher physical activity compared to those with low KOOS12 score or with informal education, respectively. There were less participants aged over 75 years of age in the Malay group, compared to other groups (*p*= 0.002). Further details of cross tabulations are presented in Tables S5 & S6 in Additional file 1.

**Table 4:** Ordinal logistic regression analyses for detriments of physical activity

Determinant	Inactive or low physical activity level, n (%)	Bi-variable analysis			Multivariable analysis		
		OR	95% CI	*P* value	Adj. OR	95% CI	*P* value
**Age in years**							
≤ 54	41 (71.93)	Ref	-	-	Ref	-	-
55-64	139 (75.54)	0.74	0.40 to 1.37	0.335	-	-	-
65-74	97 (68.31)	1.17	0.63 to 2.19	0.623	-	-	-
≥75	34 (82.93)	0.40	0.17 to 0.93	0.033	0.29	0.14 to 0.60	0.001
**Gender**							
Male	83 (61.94)	Ref	-	-	Ref	-	-
Female	227 (78.55)	0.44	0.29 to 0.67	<0.001	0.46	0.29 to 0.73	0.001
**BMI**							
Healthy - <23 kg/m^2^	58 (70.73)	Ref	-	-	Ref	-	-
Overweight - 23-27.4 kg/m^2^	128 (67.72)	1.29	0.77 to 2.18	0.333	-	-	-
Obesity - ≥ 27.5 kg/m^2^	112 (82.96)	0.50	0.28 to 0.88	0.016	0.47	0.30 to 0.74	0.001
**Ethnicity**							
Chinese	237 (71.39)	Ref	-	-	Ref	-	-
Malay	36 (85.71)	0.37	0.19 to 0.72	0.004	0.44	0.22 to 0.88	0.021
Indian	33 (76.74)	0.92	0.49 to 1.70	0.778	-	-	-
Others	5 (71.43)	0.96	0.20 to 4.70	0.961	-	-	-
**Employment status**							
Employed	177 (75.32)	Ref	-	-	-	-	-
Unemployed	12 (63.16)	1.06	0.40 to 2.84	0.909	-	-	-
Homemaker	39 (81.25)	0.80	0.43 to 1.47	0.469	-	-	-
Retired	80 (68.97)	1.10	0.70 to 1.72	0.689	-	-	-
**Education level**							
Informal	12 (75.00)	Ref	-	-	Ref	-	-
Primary	51 (79.69)	1.06	0.34 to 3.33	0.918	-	-	-
Secondary	164 (77.00)	1.11	0.38 to 3.23	0.841	-	-	-
Diploma	41 (64.06)	2.59	0.83 to 8.09	0.101	1.85	1.05 to 3.27	0.032
University	30 (58.82)	3.13	0.99 to 9.96	0.053	2.26	1.23 to 4.15	0.011
Others	13 (81.25)	1.37	0.33 to 5.79	0.665	-	-	-
**Charlson comorbidity index,** mean ± SD	311 (73.35)	0.92	0.58 to 1.45	0.709	-	-	-
**KOOS-12**							
1 Quartile (<54.17)	81 (81.82)	Ref	-	-	-	-	-
2 Quartile (54.17-64.57)	75 (76.53)	1.19	0.67, 2.12	0.482	-	-	-
3 Quartile (64.58-74.99)	83 (76.15)	1.33	0.76, 2.32	0.269	-	-	-
4 Quartile (>75)	72 (61.02)	2.65	1.54, 4.56	<0.001	1.98	1.26 to 3.13	0.003

*Ref *Reference group

## Discussion

This study offers novel insights into self-reported physical activity levels, and its correlates, for a multi-cultural Asian population of people with knee osteoarthritis who are seeking care. Most participants were categorized as having low physical activity levels, and those with obesity, from the eldest age category (i.e., ≥75), or who identified as Malay or female, were less physically active than those with a healthy BMI, younger (<54 years old), Chinese and males, respectively. We observed a significant, but weak, positive correlation between physical activity level and function, and statistically significant differences in function between those categorized as having moderate physical activity levels, compared to those who are either inactive or who have low physical activity levels. However, differences in function between groups may not be clinically important. We also observed significant, but weak, correlations between physical activity level and either kinesiophobia, self-efficacy or quality of life. Further exploration of these relationships is warranted to identify whether interventions targeting factors such as kinesiophobia and self-efficacy improve physical activity, or whether improvements in physical activity reduces kinesiophobia and improves quality of life. Understanding this further has great potential to facilitate creation of cost-effective, non-surgical interventions to improve symptoms, function, quality of life and health for people with knee osteoarthritis.

### Inadequate physical activity levels of an Asian population seeking care for knee osteoarthritis

Consistent with research around the world [16, 51], three-quarters of participants in our study reported being either inactive or having low physical activity levels. Therefore, the majority are not optimally engaging with guideline-recommended first-line care for the management of knee osteoarthritis [12–15]. Engaging in, or increasing level of, physical activity for people with knee osteoarthrosis is recommended due to its potential to improve symptoms, function, joint health and overall health, whilst minimizing the personal and societal burdens they create [11, 52]. Healthcare professionals should therefore routinely be screening for, and offer interventions to facilitate improvements in, physical activity for people with knee osteoarthritis. However, evidence indicates that healthcare professionals are failing to implement this clinically, as many people with knee osteoarthritis fail to receive physical activity and lifestyle advice prior to orthopedic or physiotherapy consultations [53–55]. Our findings may support this notion considering that participants would have already attended a primary healthcare consultation prior to being referred to an orthopedic surgeon or physiotherapist for enrollment in the study, yet still reported low physical activity levels. A lack of knowledge, confidence, skills, time and/or resources have all been identified as potential barriers to screening for, and provision of, physical activity interventions by healthcare professionals outside of Asia [56–60]. Exploration of the barriers and enablers to the provision of physical activity interventions by healthcare professionals in Asia is warranted to improve implementation of guideline-recommended first-line care.

Participants with obesity, from the eldest age category (i.e., ≥75), or who identified as Malay or female, were less physically active than those with a healthy BMI, younger (<54 years old), or who identified as Chinese or male, respectively. These findings align to previous research for people with, and without, knee osteoarthritis [19, 61]. Future research is warranted to understand why certain sub-populations were less physically active considering that physical activity is recommended for all people with knee osteoarthritis regardless of comorbidities, age, gender or race [13]. Conscious or unconscious attitudes and biases of people with knee osteoarthritis, or by healthcare professionals, may contribute towards engagement in, or the quality and provision of, physical activity interventions. For example, weight stigma [62], ageism [63] and beliefs that osteoarthritis is a ‘wear and tear’ condition [64] are potential barriers to physical activity, and these may all contribute to lower perceived levels of physical activity. However, investigation of such factors is underexplored in Asia. We recommend that future physical activity interventions and initiatives are co-designed in collaboration with diverse groups of people with knee osteoarthritis, and healthcare professionals, to reduce implementation and engagement barriers.

### Relationship between physical activity and function, kinesiophobia, self-efficacy and quality of life

Supporting previous findings in Western and Caucasian populations with knee osteoarthritis [22, 23], lower physical activity levels of people in our study were correlated with increased self-reported function and kinesiophobia, plus decreased self-efficacy and quality of life. The relationship between physical activity and these outcomes is complex and may be partially underpinned by common inaccurate beliefs about symptoms, the condition and its management [65, 66]. For example, people with knee osteoarthritis commonly believe that; i) pain is a sign of increasing damage, ii) physical activity will ‘wear down’ their joints or, iii) that surgery is inevitable [64, 67, 68]. Such beliefs may be due to pervasive inaccurate online information about the condition and its management [69], and be exacerbated by common misconceptions of healthcare professionals [64, 70]. When viewed through a fear avoidance model lens [65], these beliefs have the potential to contribute towards decreased function, catastrophizing of symptoms, kinesiophobia and a decrease in one’s self-efficacy for, and engagement in, physical activity. This in turn may reduce an individual with knee osteoarthritis’ quality of life.

Combining education with physical activity/exercise therapy interventions is more effective than providing the physical activity/exercise therapy intervention alone for improving physical activity, symptoms, self-efficacy, psychological distress and quality of life for people with knee osteoarthritis [32, 71–73]. Future physical activity interventions for people with knee osteoarthritis are therefore encouraged to include education to empower positive attitudes and behaviors towards active lifestyles, including dispelling common misconceptions that physical activity or exercise therapy is unsafe or harmful [67].

Additionally, initiatives to improve knowledge of, and promote screening for, psychological factors such kinesiophobia and self-efficacy by healthcare professionals is encouraged. This will allow the early identification of higher risk, psychologically vulnerable patients and facilitate the provision of targeted interventions to address these psychological factors and subsequent reductions in physical activity.

### Limitations and future directions

To our knowledge this is the first investigation of self-reported physical activity and its possible correlates for an Asian population with knee osteoarthritis. The large proportion of people with knee osteoarthritis who self-report inactivity or low levels of physical activity is consistent with previous literature in Western populations [16, 51] and should be cause for concern. However, our results may not be representative of all Asian communities with knee osteoarthritis. For example, we cannot assume that all Malay communities throughout Asia will be less physically active compared to Chinese communities. Our findings are therefore limited to Singapore’s highly urbanized and multiethnic population. It is also important to note that although the UCLA activity score is widely used in knee osteoarthritis research and has been demonstrated to have a strong correlation to average steps per day [27, 74], it is not an objective measure of physical activity. Therefore, there may be a discrepancy between participants perceived and actual physical activity. Additionally, recruitment to our study occurred during the COVID-19 pandemic when rates of inactivity may have been at their highest due to national level restrictions [75]. Future research involving both self-reported and objective measures of physical activity may be required to confirm our findings at a time when COVID-19 restrictions are no longer in enforcement. We did not identify the presence, or stage, of radiographic osteoarthritis for enrolled participants. Although conflicting evidence exists regarding stage of radiographic osteoarthritis and its association to outcomes including pain, function and physical activity [76–80], we cannot determine whether this was an important factor, or correlate of physical activity, for our study population. Future research is encouraged to explore the relationship of radiographic osteoarthritis and physical activity levels within Asian populations.

Due to the nature of our study, we cannot determine whether lower self-reported physical activity levels cause/result in worse self-reported outcomes, or whether worse self-reported outcomes cause/result in lower self-reported physical activity levels. The collection of longitudinal data is encouraged to determine any relationship between, and changes within, physical activity level and outcomes such as kinesiophobia, self-efficacy and quality of life. It is also important to note that this study only explored person-level correlates of physical activity. However, social and environmental factors are also known to influence physical activity levels [81]. Research investigating social and environmental correlates of physical activity are again under explored with Asian populations [82] and should be prioritised in future.

## Conclusion

Three quarters of people with knee osteoarthritis seeking care in our study reported being inactive or having low physical activity levels. Future research is encouraged to understand barriers and enablers to increasing physical activity from both an individual with knee osteoarthritis’ and from a healthcare professional's viewpoint. Co-designing and implementing interventions to increase engagement in physical activity has great potential to improve symptoms, function, health, and quality of life for the majority of people with knee osteoarthritis.

### Supplementary Information

**Additional file 1:Table S1.** Summarised participant characteristics and UCLA data for all participants. **Table S2. **Ordinal UCLA activity score and knee osteoarthritis outcome score (KOOS-12). **Table S3.** Ordinal UCLA activity score and brief fear of movement (BFOM). **Table S4. **Ordinal UCLA activity score and EQ-5D-5L. **Table S5. **Cross tabulation for race and age (Fisher Extract test). **Table S6. **Cross tabulation for gender and age (Pearson chi-squared test).

## Data Availability

The datasets used and/or analyzed during the current study are available from the corresponding author on reasonable request.

## Abbreviations

ASES: Arthritis self-efficacy scale
BFOM: Brief fear of movement
BMI: Body Mass Index
KOOS: Knee Osteoarthritis Outcome Score
