# Supplementary material for: A cross sectional study exploring the relationship of self-reported physical activity with function, kinesiophobia, self-efficacy and quality of life in an Asian population seeking care for knee osteoarthritis
Source: BMC Musculoskelet Disord. 2024 Jan 18;25:74. doi: 10.1186/s12891-024-07181-y (PMC10795260; doi:10.1186/s12891-024-07181-y)
Supplement: Supplementary file 1 — Additional file 1:Table S1. Summarised participant characteristics and UCLA data for all participants. Table S2. Ordinal UCLA activity score and knee osteoarthritis outcome score (KOOS-12). Table S3. Ordinal UCLA activity score and brief fear of movement (BFOM). Table S4. Ordinal UCLA activity score and EQ-5D-5L. Table S5. Cross tabulation for race and age (Fisher Extract test). Table S6. Cross tabulation for gender and age (Pearson chi-squared test). [file 12891_2024_7181_MOESM1_ESM.pdf]

Additional file 1

Table S1. Summarised participant characteristics and UCLA data for all participants

**Characteristics**		**UCLA activity score, median (IQR)**	**UCLA in category, n (%)**				
			**Inactive**	**Low**	**Moderate**	**High**	**Very high**
Overall		4 (3, 5)	46 (10.8)	265 (62.5)	92 (21.7)	16 (3.8)	5 (1.2)
Age	≤ 54	4 (3, 5)	6 (13.0)	35 (13.2)	9 (9.8)	3 (18.8)	4 (80.0)
	55-64	4 (3, 4)	24 (52.2)	115 (43.4)	36 (39.1)	8 (50.0)	1 (20.0)
	65-74	4 (3, 5)	7 (15.2)	90 (34.0)	40 (43.5)	5 (31.3)	0 (0.0)
	≥75	2 (3, 4)	9 (19.6)	25 (9.4)	7 (7.6)	0 (0.0)	0 (0.0)
Gender	Male	4 (3, 5)	10 (21.7)	73 (27.7)	37 (40.2)	10 (62.5)	4 (80.0)
	Female	4 (3, 4)	36 (78.3)	191 (72.3)	55 (59.8)	6 (37.5)	1 (20.0)
Ethnicity	Chinese	4 (3, 5)	33 (71.7)	204 (77.0)	76 (82.6)	15 (93.8)	4 (80.0)
	Malay	3 (3, 4)	10 (21.7)	26 (9.8)	5 (5.4)	0 (0.0)	1 (20.0)
	Indian	4 (3, 4)	2 (4.3)	31 (11.7)	10 (10.9)	0 (0.0)	0 (0.0)
	Others	4 (3, 6)	1 (2.2)	4 (1.5)	1 (1.1)	1 (6.3)	0 (0.0)
BMI	Healthy	4 (3, 5)	8 (18.6)	50 (19.4)	24 (26.4)	2 (12.5)	1 (20.0)
	Overweight	4 (3, 5)	11 (25.6)	118 (45.7)	47 (51.6)	12 (75.0)	3 (60.0)
	Obesity	3 (3, 4)	24 (55.8)	90 (34.9)	20 (22.0)	2 (12.5)	1 (20.0)
Employment status	Employed	4 (3, 4)	21 (46.7)	156 (59.3)	46 (51.7)	8 (50.0)	4 (80.0)
	Unemployed	4 (3, 5)	4 (8.9)	8 (3.0)	6 (6.7)	1 (6.3)	0 (0.0)
	Homemaker	4 (3, 4)	4 (8.9)	35 (13.3)	9 (10.1)	0 (0.0)	0 (0.0)
	Retired	4 (3, 5)	16 (35.6)	64 (24.3)	28 (31.5)	7 (43.8)	1 (20.0)
Education level	Informal	3 (3, 4.5)	3 (6.5)	9 (3.4)	4 (4.3)	0 (0.0)	0 (0.0)
	PSLE	4 (3, 4)	8 (17.4)	43 (16.2)	12 (13.0)	1 (6.3)	0 (0.0)
	Secondary	4 (3, 4)	30 (65.2)	134 (50.6)	44 (47.8)	5 (31.3)	0 (0.0)
	Diploma	4 (3, 5)	3 (6.5)	38 (14.3)	15 (16.3)	6 (37.5)	2 (40.0)
	University	4 (3, 6)	1 (2.2)	29 (10.9)	16 (17.4)	3 (18.8)	2 (40.0)
	Others	3.5 (3, 4)	1 (2.2)	12 (4.5)	1 (1.1)	1 (6.3)	1 (20.0)

Table S2. Ordinal UCLA activity score and knee osteoarthritis outcome score (KOOS-12)

Multiple Comparisons

Dependent Variable: koos12 score

Bonferroni

(I) ucla_5cat	(J) ucla_5cat	Mean Difference (I-J)	Std. Error	Sig.	95% Confidence Interval Lower Bound	95% Confidence Interval Upper Bound
Inactive	Low physical activity level	-3.97246	2.49922	1.000	-11.0252	3.0803
	Moderate physical activity level	-10.19033*	2.82549	.003	-18.1638	-2.2169
	High physical activity level	-8.39554	4.54133	.652	-21.2111	4.4200
	Very high physical activity level	-15.24404	7.36796	.392	-36.0363	5.5482
Low physical activity level	Inactive	3.97246	2.49922	1.000	-3.0803	11.0252
	Moderate physical activity level	-6.21787*	1.89341	.011	-11.5610	-.8747
	High physical activity level	-4.42308	4.02807	1.000	-15.7902	6.9440
	Very high physical activity level	-11.27158	7.06318	1.000	-31.2037	8.6606
Moderate physical activity level	Inactive	10.19033*	2.82549	.003	2.2169	18.1638
	Low physical activity level	6.21787*	1.89341	.011	.8747	11.5610
	High physical activity level	1.79478	4.23823	1.000	-10.1654	13.7550
	Very high physical activity level	-5.05372	7.18511	1.000	-25.3299	15.2225
High physical activity level	Inactive	8.39554	4.54133	.652	-4.4200	21.2111
	Low physical activity level	4.42308	4.02807	1.000	-6.9440	15.7902
	Moderate physical activity level	-1.79478	4.23823	1.000	-13.7550	10.1654
	Very high physical activity level	-6.84850	8.01661	1.000	-29.4712	15.7742
Very high physical activity level	Inactive	15.24404	7.36796	.392	-5.5482	36.0363
	Low physical activity level	11.27158	7.06318	1.000	-8.6606	31.2037
	Moderate physical activity level	5.05372	7.18511	1.000	-15.2225	25.3299
	High physical activity level	6.84850	8.01661	1.000	-15.7742	29.4712

*. The mean difference is significant at the 0.05 level.

Table S3. Ordinal UCLA activity score and brief fear of movement (BFOM)

Multiple Comparisons

Dependent Variable: bfom score

Bonferroni

(I) ucla_5cat	(J) ucla_5cat	Mean Difference (I-J)	Std. Error	Sig.	95% Confidence Interval Lower Bound	95% Confidence Interval Upper Bound
Inactive	Low physical activity level	1.773	.741	.174	-.32	3.87
	Moderate physical activity level	3.180*	.921	.006	.57	5.79
	High physical activity level	2.611	1.520	.869	-1.69	6.91
	Very high physical activity level	4.389	2.451	.744	-2.55	11.32
Low physical activity level	Inactive	-1.773	.741	.174	-3.87	.32
	Moderate physical activity level	1.406	.689	.421	-.54	3.36
	High physical activity level	.838	1.391	1.000	-3.10	4.77
	Very high physical activity level	2.616	2.373	1.000	-4.10	9.33
Moderate physical activity level	Inactive	-3.180*	.921	.006	-5.79	-.57
	Low physical activity level	-1.406	.689	.421	-3.36	.54
	High physical activity level	-.568	1.495	1.000	-4.80	3.66
	Very high physical activity level	1.209	2.435	1.000	-5.68	8.10
High physical activity level	Inactive	-2.611	1.520	.869	-6.91	1.69
	Low physical activity level	-.838	1.391	1.000	-4.77	3.10
	Moderate physical activity level	.568	1.495	1.000	-3.66	4.80
	Very high physical activity level	1.778	2.719	1.000	-5.92	9.47
Very high physical activity level	Inactive	-4.389	2.451	.744	-11.32	2.55
	Low physical activity level	-2.616	2.373	1.000	-9.33	4.10
	Moderate physical activity level	-1.209	2.435	1.000	-8.10	5.68
	High physical activity level	-1.778	2.719	1.000	-9.47	5.92

*. The mean difference is significant at the 0.05 level.

Table S4. Ordinal UCLA activity score and EQ-5D-5L

Multiple Comparisons

Dependent Variable: eq5d index score

Dunnett C

(I) ucla_5cat	(J) ucla_5cat	Mean Difference (I-J)	Std. Error	95% Confidence Interval Lower Bound	95% Confidence Interval Upper Bound
Inactive	Low physical activity level	-.14361	.06579	-.3300	.0428
	Moderate physical activity level	-.24455*	.06826	-.4379	-.0512
	High physical activity level	-.18881	.11246	-.5274	.1497
	Very high physical activity level	-.41743*	.06679	-.6188	-.2160
Low physical activity level	Inactive	.14361	.06579	-.0428	.3300
	Moderate physical activity level	-.10095*	.03215	-.1900	-.0118
	High physical activity level	-.04520	.09499	-.3373	.2469
	Very high physical activity level	-.27383*	.02890	-.3817	-.1660
Moderate physical activity level	Inactive	.24455*	.06826	.0512	.4379
	Low physical activity level	.10095*	.03215	.0118	.1900
	High physical activity level	.05574	.09672	-.2408	.3522
	Very high physical activity level	-.17288*	.03415	-.2915	-.0543
High physical activity level	Inactive	.18881	.11246	-.1497	.5274
	Low physical activity level	.04520	.09499	-.2469	.3373
	Moderate physical activity level	-.05574	.09672	-.3522	.2408
	Very high physical activity level	-.22862	.09568	-.5310	.0737
Very high physical activity level	Inactive	.41743*	.06679	.2160	.6188
	Low physical activity level	.27383*	.02890	.1660	.3817
	Moderate physical activity level	.17288*	.03415	.0543	.2915
	High physical activity level	.22862	.09568	-.0737	.5310

*. The mean difference is significant at the 0.05 level.

Table S5 – Cross tabulation for race and age (Fisher Extract test)

	Ethnicity, n (%)				
	Chinese	Malay	Indian	Others	P value
**Age**					
≤ 54	38 (11.4)	9 (21.4)	9 (20.9)	1 (14.3)	0.002
55-64	135 (40.5)	24 (57.1)	22 (51.2)	4 (57.1)	
65-74	127 (38.1)	7 (16.7)	8 (18.6)	0 (0.0)	
≥75	33 (9.91)	2 (4.8)	4 (9.3)	2 (28.6)	

Table S6 – Cross tabulation for gender and age (Pearson chi-squared test)

	Gender, n (%)		
	Male	Female	P value
**Age**			
≤ 54	14 (10.5)	43 (14.8)	0.105
55-64	56 (41.8)	129 (44.5)	
65-74	45 (33.5)	97 (33.5)	
≥75	19 (14.2)	21 (7.2)	
